# Policy cognition, perceived stress, coping dimensions, and adolescent health behaviors under physical education examination reform: a cross-sectional SEM study

**DOI:** 10.3389/fpsyg.2026.1896758

**Published:** 2026-09-22

**Authors:** Mengqiang Hu, Weimiao Wu, Shihong Zhang, Ying He, Jinghang Li, Shunming Huang

**Affiliations:** 1 School of Physical Education, Woosuk University, Jeonju, Republic of Korea; 2 School of Marxism, Qingdao Binhai University, Qingdao, China; 3 College Sports and Health, Huaihua University, Huaihua, Hunan, China; 4 School of Humanities, Henan University of Science and Technology, Luoyang, China; 5 Resort Branch of the Third Middle School of Kunming City China, Kunming, Yunnan, China

**Keywords:** adolescents, avoidance-oriented coping, emotion-oriented coping, health behaviors, perceived stress, policy cognition, structural equation modeling, task-oriented coping

## Abstract

**Background:**

Physical education is increasingly embedded in high-stakes entrance examinations in China. However, the cross-sectional associations among adolescents' policy cognition, perceived stress, distinct coping dimensions, and health behaviors remain insufficiently understood.

**Methods:**

A cross-sectional questionnaire survey was conducted among 1,513 junior secondary students enrolled in one participating school in Yunnan Province, China. Rural–urban background was measured as an individual-level self-reported characteristic and did not represent the location or institutional classification of the participating school. Task-oriented, emotion-oriented, and avoidance-oriented coping were modeled as three correlated but distinct constructs. Five documented health-behavior outcomes were analyzed: physical activity, dietary habits, weekday sleep duration, screen-time behavior, and overall routine regularity. Sensitivity analyses adjusted first for sex and rural–urban background and then for PE talent status, school PE lessons, extracurricular physical activity, family exercise frequency, and parental encouragement.

**Results:**

A correlated three-factor coping model fitted substantially better than a one-factor model. In the fully adjusted model involving 1,198 participants, perceived stress was negatively associated with physical activity (β = −0.246), dietary habits (β = −0.167), weekday sleep duration (β = −0.167), screen-time health score (β = −0.183), and overall routine regularity (β = −0.164). Task-oriented coping was positively associated with the five outcomes (β = 0.254, 0.174, 0.157, 0.222, and 0.192, respectively; all *p* < 0.001). Emotion-oriented coping was not independently associated with the outcomes, whereas avoidance-oriented coping was negatively associated with dietary habits (β = −0.213, *p* < 0.001). Direct associations between policy cognition and most health outcomes were attenuated after extended covariate adjustment.

**Conclusions:**

Perceived stress and task-oriented coping were the most consistent cross-sectional correlates of the documented health-behavior outcomes. The results are associational and do not establish temporal ordering, causal mediation, or intervention effectiveness.

## 1 Background

High-stakes examinations are embedded in students' daily lives and are associated with experiences extending beyond academic outcomes. In China, recent reforms have increased the weight of physical education (PE) in high school entrance examinations and promoted workload reduction and “Healthy China 2030” initiatives (Central Committee of the Communist Party of China, 2016). Recent evidence has begun to examine potential health impacts of the PE entrance-examination policy among middle school students (Compas et al., 2017). These reforms are intended to counteract physical inactivity and health risks among adolescents (Cohen et al., 1983; Guthold et al., 2020; Liu et al., 2018; Lu et al., 2017; Ntoumanis et al., 2021; World Health Organization., 2018, 2020; Inchley et al., 2020; Janssen and LeBlanc, 2010) by making physical fitness and health behaviors more salient in school accountability and student assessment. However, healthier routines do not necessarily accompany policy change, and the observed associations may depend on how students interpret policy signals and report managing examination-related stress. Associations between policy reform and students' health-related routines may vary according to how students perceive and interpret policy signals and report managing stress in exam-oriented environments.

Educational psychology research indicates that students' cognitive appraisals of school demands and expectations are critical for motivation, wellbeing, and self-regulation (Deci and Ryan, 2000; Hagger and Chatzisarantis, 2016; Ntoumanis et al., 2021; Standage et al., 2012; Teixeira et al., 2012; Vasconcellos et al., 2020). When requirements are perceived as legitimate, fair, and aligned with personal values, students are more likely to internalize them and engage in adaptive behaviors. Conversely, when demands are seen as arbitrary or punitive, they can trigger stress, disengagement, and maladaptive coping.

In the present study, policy cognition is defined as adolescents' cognitive and evaluative representation of the PE examination policy: what they understand about its rules and scoring standards, whether they regard it as reasonable and necessary, and whether they perceive adequate school guidance and long-term health value. It therefore refers neither to objective policy exposure nor merely to policy awareness or behavioral compliance. This definition is informed by policy feedback theory, which proposes that enacted policies provide information and interpretive cues that was associated with how target groups understand and evaluate policy, and by policy-design research showing that policy messages can be absorbed and interpreted by the populations to whom they are directed (Mettler and Soss, 2004; Pierson, 1993; Schneider and Ingram, 1993). Differences in policy cognition may therefore help explain why some students respond to PE examination reform with sustained physical activity and health-promoting routines, whereas others experience pressure, uncertainty, or avoidance.

Self-determination theory (SDT) emphasizes that internalization of external demands depends on support for basic psychological needs of autonomy, competence, and relatedness. When social contexts are autonomy-supportive and need-supportive, external regulations can be transformed into personally endorsed values and goals (Bocarro et al., 2012; Carson et al., 2016; Dong et al., 2023; Hale and Guan, 2015; Poitras et al., 2016; Yunnan Provincial Department of Education, 2020). Existing work in PE has focused largely on classroom-level factors such as autonomy-supportive teaching and need-supportive climates. Building on these perspectives, policy cognition is treated not as a direct measure of self-determination-theory internalization but as a policy-specific subjective appraisal. More favorable policy appraisals may co-occur with greater perceived meaningfulness of policy requirements, whereas less favorable appraisals may co-occur with greater reported stress. These theoretically specified associations do not establish that policy cognition causes internalization, stress, coping, or health behavior.

Building on these perspectives, we treat policy cognition not as a direct measure of self-determination theory internalization, but as a policy-specific appraisal that may facilitate or hinder such internalization. When adolescents perceive the PE examination policy as clear, fair, necessary, and genuinely health-oriented, they may be more likely to regard its requirements as meaningful and to integrate them into their personal goals and daily routines (Carson et al., 2016; Hale and Guan, 2015; Hu and Bentler, 1999; Kline, 2016; Poitras et al., 2016; Schreiber et al., 2006). Conversely, less favorable appraisals of rule clarity, reasonableness, guidance, or health value, or a narrowly performance-oriented policy may impede internalization and intensify stress-related responses.

At the same time, exam-oriented environments are inherently stressful. Stress–coping frameworks highlight that individuals' appraisals of demands and their coping responses are closely related to emotional responses and behavioral outcomes (Deci and Ryan, 2000; Ryan and Deci, 2017, 2000; Schneider and Ingram, 1993; Lazarus and Folkman, 1984; Endler and Parker, 1990; Skinner and Zimmer-Gembeck, 2007). Adolescents facing PE and academic examinations may report high levels of perceived stress, which has been associated with less favorable physical activity, sleep, and self-care patterns (Sun et al., 2013; Zhu et al., 2021; Steare et al., 2023). Yet students differ in how they respond to stress.

Adolescents may use task-oriented, emotion-oriented, and avoidance-oriented coping when facing PE examination and academic demands (Lazarus and Folkman, 1984; Endler and Parker, 1990; Skinner and Zimmer-Gembeck, 2007). These coping dimensions are conceptually distinct and should not be assumed to have equivalent associations with adolescent health behavior. Task-oriented coping involves planning, problem solving, time management, goal focus, and active resource seeking. Emotion-oriented coping reflects emotional dysregulation, self-blame, irritability, discouragement, and withdrawal in response to negative affect. Avoidance-oriented coping reflects cognitive distraction and behavioral disengagement from examination-related demands. Accordingly, the revised analysis treats the three coping dimensions separately rather than representing them as indicators of a single uniformly positive coping construct.

Adolescent development is also embedded in broader social ecologies. Social-ecological models emphasize that individual behavior is associated with contextual characteristics across school, family, and broader policy environments (Hagger and Chatzisarantis, 2016; Standage et al., 2012; Sallis et al., 2015; Bronfenbrenner and Morris, 2006). Accordingly, sex, rural–urban background, school PE opportunities, previous physical-activity participation, and family support were considered as potential contextual covariates in the revised analyses.

Despite rich literatures on motivation, stress, and health behaviors, three gaps remain. First, most research on educational policies and high-stakes examinations has examined outcomes such as achievement or test anxiety, while relatively few studies have focused on health behaviors as a central outcome in policy evaluation. Second, work on PE and health behaviors has largely emphasized classroom-level or teacher-level processes (e.g., autonomy-supportive teaching), with comparatively little attention to how students interpret formal policy schemes and how these interpretations are associated with micro-level behaviors within the context of macro-level reforms.

Third, previous studies have frequently represented coping and health behavior using broad composite constructs. Less attention has been paid to whether task-oriented, emotion-oriented, and avoidance-oriented coping show different associations with specific health-behavior outcomes, or whether these associations remain after adjustment for relevant demographic, school, activity-related, and family characteristics.

### 1.1 Policy cognition as a policy-related subjective appraisal

In the present study, policy cognition is conceptualized as adolescents' subjective understanding and evaluation of a specific PE examination policy. It encompasses perceived rule clarity, reasonableness and necessity, preparedness and school guidance, perceived health value, and social salience. Policy cognition is treated as a policy-related psychological correlate that may be statistically associated with perceived stress, coping dimensions, and health behaviors.

The construct is not intended to represent objective policy knowledge, implementation intensity, or a direct measure of self-determination-theory internalization. Rather, it represents a context-specific, satisfaction-based appraisal of the PE entrance-examination policy. Because all variables were measured concurrently, the proposed ordering represents a theory-informed statistical specification rather than an established temporal or causal process.

### 1.2 The present study

First, how was policy cognition associated with perceived stress and task-oriented, emotion-oriented, and avoidance-oriented coping? Second, how were perceived stress and the three coping dimensions associated with physical activity, dietary habits, weekday sleep duration, screen-time behavior, and overall routine regularity? Third, did these associations remain similar after adjustment for available demographic, school-PE, prior-activity, and family-related covariates?

Because all variables were measured concurrently, these research questions concern statistical associations rather than temporal or causal processes. Figure 1 presents the revised cross-sectional structural model.

**Figure 1:**
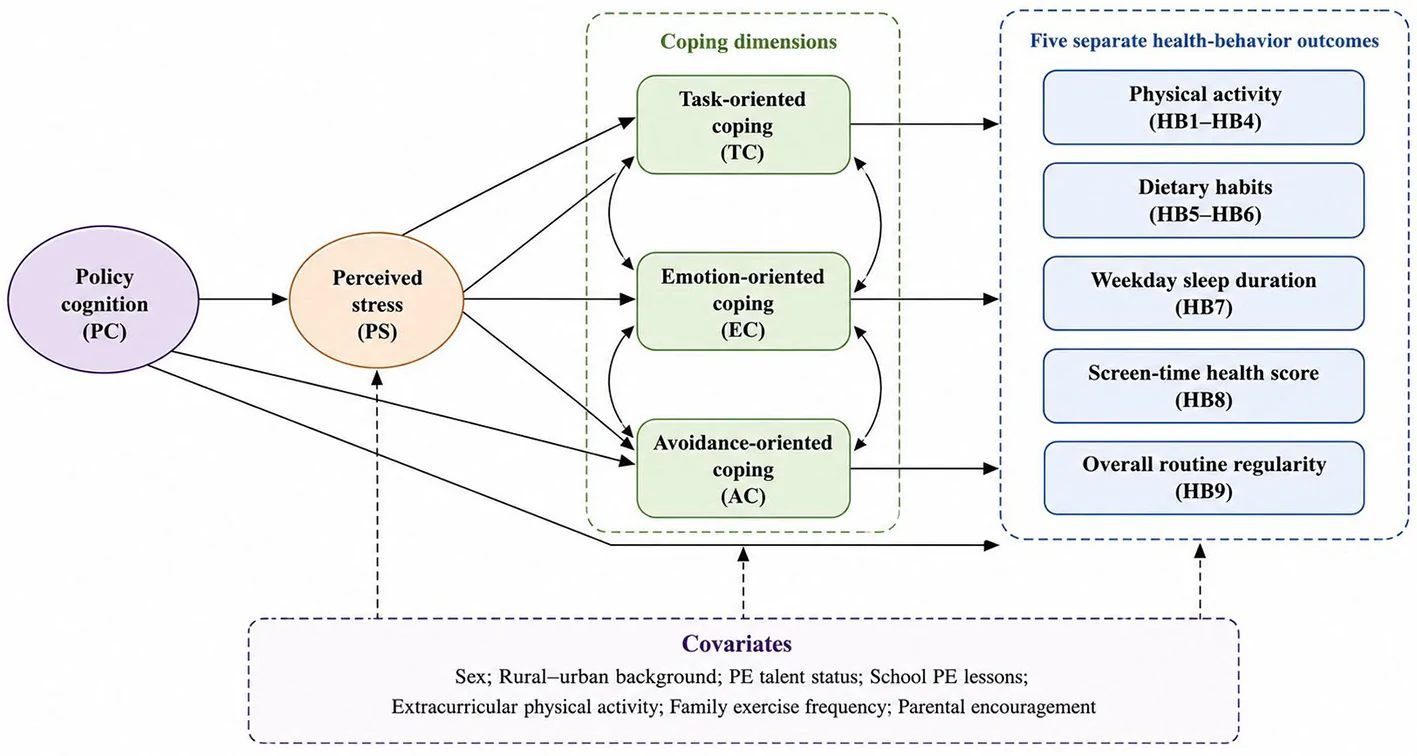
Revised cross-sectional structural model of policy cognition, perceived stress, coping dimensions, and five health-behavior outcomes. Solid arrows represent specified concurrent regression associations. Arrows entering the health-outcome block represent separately estimated paths to physical activity, dietary habits, weekday sleep duration, screen-time health score, and overall routine regularity. Dashed arrows indicate that sex, rural–urban background, PE talent status, school PE lessons, extracurricular physical activity, family exercise frequency, and parental encouragement were included as covariates included in the equations for perceived stress, the coping dimensions, and the health-behavior outcomes, the coping dimensions, and the health-behavior outcomes. Curved double-headed arrows indicate covariances among the coping dimensions. The diagram does not establish temporal or causal direction.

## 2 Methods

### 2.1 Participants

Data were obtained from a cross-sectional, school-based questionnaire survey conducted in one participating junior secondary school in Yunnan Province, China. A total of 1,513 students provided valid responses, including 748 boys (49.4%) and 765 girls (50.6%).

Rural–urban background was measured as an individual-level self-reported demographic characteristic and was coded as 1 = rural and 2 = urban. A total of 724 students (47.9%) reported a rural background, and 789 students (52.1%) reported an urban background. These categories describe students' reported backgrounds and do not classify the participating school as either a rural or an urban school.

Eligible participants were enrolled in junior secondary education and were able to complete the Chinese-language questionnaire independently. Only students for whom written informed consent had been obtained from a parent or legal guardian and who provided personal assent before questionnaire completion were eligible to participate. Students who were absent during data collection or had extensive missing data on the focal study variables were excluded.

Verified class identifiers were not retained in the de-identified analysis-ready dataset. Consequently, the exact number of participating classes and the student-to-class nesting structure could not be reconstructed retrospectively.

### 2.2 Measures

#### 2.2.1 Policy cognition

Policy cognition was assessed using a five-item, context-specific scale developed for the present study. The items were mapped to the operational definition presented in Section 1.1 and to the documented content of the Yunnan PE entrance-examination policy (Boateng et al., 2018; Yunnan Provincial Department of Education, 2020). No retained documentation was available to verify a formal expert-panel review, cognitive interviewing, or an independent pilot study. The measure should therefore be regarded as a preliminary, theory-informed, and policy-specific instrument rather than as an established generic scale.

The five items represented five related content facets: rule clarity; perceived reasonableness and necessity; personal preparedness and school guidance; perceived health and developmental value; and social salience. All items were rated from 1 (very dissatisfied) to 7 (very satisfied). Consequently, the mean score represented a favorable satisfaction-based subjective appraisal of the policy rather than objective policy knowledge, factual accuracy, or a direct measure of perceived fairness. The exact wording, content facets, response options, and standardized loadings are provided in Supplementary Table S1.

Item scores were averaged, with higher scores indicating a more favorable policy appraisal. In the present sample, Cronbach's α was 0.882, composite reliability was 0.886, and average variance extracted was 0.609.

#### 2.2.2 Perceived stress

Perceived stress was measured using the 10-item Simplified Chinese version of the Perceived Stress Scale (PSS-10; Chen et al., 2024; Cohen et al., 1983; Lu et al., 2017). Previous psychometric evaluation of the Simplified Chinese PSS-10 supported a two-factor structure, good internal consistency (Cronbach's α = 0.85), acceptable 2-week test–retest reliability (*r* = 0.70), and concurrent validity through significant associations with measures of depression and anxiety (Mettler and Soss, 2004). More directly relevant to the present population, a subsequent validation study involving 977 Chinese middle-school students supported a robust two-factor structure, acceptable reliability, significant associations with general mental health, self-esteem, and resilience, and measurement invariance across gender and left-behind status (Igolkina and Meshcheryakov, 2020; Chen et al., 2024).

The items asked how often, during the previous month, students felt that situations in their lives were unpredictable, uncontrollable, or overwhelming. Responses were made on a 5-point scale ranging from 1 (never) to 5 (always). Positively worded items were reverse-coded, and the mean score was calculated, with higher scores indicating greater perceived stress. In the present sample, the PSS-10 demonstrated high internal consistency (Cronbach's α = 0.910).

#### 2.2.3 Coping dimensions

Coping was assessed using 15 contextualized items representing task-oriented, emotion-oriented, and avoidance-oriented coping, based on the Coping Inventory for Stressful Situations framework (Compas et al., 2017; Lazarus and Folkman, 1984; Endler and Parker, 1990; Skinner and Zimmer-Gembeck, 2007), with five items per dimension. Students indicated how often they used each response on a five-point scale ranging from 1 (never) to 5 (almost always). The exact wording, response options, and dimensional assignment of the 15 items are presented in Supplementary Table S2.

Task-oriented coping assessed planning, problem solving, time management, goal focus, and active resource seeking. Emotion-oriented coping assessed emotional dysregulation, self-blame, irritability, discouragement, and withdrawal from training because of negative affect. Avoidance-oriented coping assessed cognitive distraction, screen-based escape, avoidance of PE classes or training, social distraction, and avoidance of examination-related topics.

The three dimensions were modeled as correlated but distinct latent constructs. Dimension-specific standardized loading ranges and reliability and convergent-validity indices are reported in Table 1.

**Table 1:** Reliability and convergent validity of coping dimensions.

Dimension	Standardized loading range	Cronbach's α	CR	AVE
Task-oriented coping	0.648–0.858	0.870	0.872	0.579
Emotion-oriented coping	0.758–0.855	0.898	0.898	0.638
Avoidance-oriented coping	0.724–0.771	0.861	0.862	0.555

#### 2.2.4 Health-behavior outcomes

Health behaviors were assessed using nine documented questionnaire items. Four items assessed the frequency and duration of moderate- and vigorous-intensity physical activity during the previous seven days. Two items assessed vegetable consumption and sugar-sweetened beverage consumption. One item assessed weekday sleep duration, one assessed daily electronic-device use, and one assessed the overall regularity of dietary and daily routines.

An additional field labeled Hb10 was present in the archived analytic dataset. However, no corresponding administered questionnaire item, wording, response options, scoring rule, or documented source could be verified. Hb10 was therefore excluded from all revised analyses, and no item content was reconstructed retrospectively.

Physical activity was calculated as the mean of HB1–HB4. Dietary habits were calculated from HB5 and the archived health-oriented analysis field for HB6. HB7 was analyzed as weekday sleep duration, HB8 as a screen-time health score, and HB9 as overall routine regularity. HB6 and HB8 were analyzed using the health-oriented values retained in the final analysis-ready dataset; no additional reverse-scoring step was applied during the revised reanalysis.

The exact wording, original response options, analysis-ready scoring direction, and outcome assignment of all nine documented health-behavior items are reported in Supplementary Table S3.

A one-factor model for HB1–HB9 was compared with a multidomain model specifying physical activity using HB1–HB4, dietary habits using HB5–HB6, and a provisional sleep/screen factor using HB7–HB8, with HB9 retained as a separate observed routine-regularity outcome (Wilhite et al., 2023; Malik et al., 2013; Paruthi et al., 2016; Wang et al., 2018; Tremblay et al., 2016). Because HB7 and HB8 represented substantively distinct behaviors and the provisional two-indicator factor was not retained, weekday sleep duration and screen-time health score were analyzed separately. The revised primary SEM treated physical activity, dietary habits, weekday sleep duration, screen-time health score, and overall routine regularity as separate outcomes.

#### 2.2.5 Covariates

Available covariates included sex, rural–urban background, PE talent status, weekly number of school PE lessons, weekly extracurricular physical activity, family exercise frequency, and parental encouragement for physical activity. Rural–urban background was coded as 1 = rural and 2 = urban.

Sex and rural–urban background were available for all 1,513 participants. The numbers of valid observations were 1,211 for PE talent status, 1,214 for weekly school PE lessons, 1,208 for extracurricular physical activity, 1,206 for family exercise frequency, and 1,212 for parental encouragement. A total of 1,198 participants had complete valid data for all extended covariates.

Grade was not retained as a valid analytic variable and was not reconstructed from birth year or other variables. Categorical covariates were represented using dummy variables in the adjusted models. Definitions, analytic representation, reference categories, and variable-specific valid sample sizes are presented in Supplementary Table S4.

### 2.3 Procedure

Data collection was conducted during regular school hours in July 2025. Before data collection, the participating school issued written administrative authorization for the research team to conduct the anonymous questionnaire survey and use the resulting de-identified data for academic research and publication. The authorization document was signed and stamped by the participating school on 9 July 2025. This document constituted school-level administrative permission and was not approval or exemption issued by an independent institutional research ethics committee.

Before the survey, parents or legal guardians received information concerning the study purpose, questionnaire procedures, voluntary nature of participation, confidentiality protections, and intended research use of the data. Written informed consent was obtained from a parent or legal guardian before the student was invited to participate.

Questionnaires were administered in classroom settings using paper-and-pencil forms. Trained research assistants who were not the students' classroom teachers explained the study purpose and procedures using age-appropriate language. Students were informed that participation was voluntary, that they could decline or discontinue participation without penalty, and that participation and questionnaire responses would not affect their grades, school evaluation, relationships with teachers, or access to school activities. Student assent was obtained before questionnaire completion.

Students completed the questionnaires individually. Research assistants were available to clarify instructions or item wording but did not suggest or comment on responses. Classroom teachers did not inspect completed questionnaires or have access to individual responses. Questionnaire completion required approximately 10–15 min.

No names, student identification numbers, telephone numbers, contact details, or other directly identifying information were collected. Completed questionnaires were collected by the research team, checked for completeness, and entered into an electronic database. The analysis dataset was de-identified and used exclusively for research purposes. Access to the electronic data was restricted to authorized members of the research team.

### 2.4 Data analysis

Preliminary data management and descriptive analyses were conducted using SPSS. The revised confirmatory factor analyses and structural equation models were estimated in Python using semopy version 2.3.11 with maximum-likelihood estimation. The final analysis-ready dataset contained complete data on the focal study variables for all 1,513 participants. No imputation was performed for missing extended covariates; the extended-adjusted model used complete-case analysis.

Because policy cognition was assessed using a newly developed, context-specific five-item instrument, additional analyses were conducted to examine its dimensionality (Fabrigar et al., 1999; Boateng et al., 2018; Horn, 1965). Sampling adequacy was evaluated using the Kaiser–Meyer–Olkin statistic and Bartlett's test of sphericity. Exploratory factor analysis was conducted using principal-axis factoring, and parallel analysis was used to determine the number of factors to retain. Because one factor was retained, no rotation was applied. The exploratory results were considered together with confirmatory factor loadings, corrected item–total correlations, Cronbach's α, composite reliability, and average variance extracted.

Alternative coping measurement models compared a one-factor model with a correlated three-factor model comprising task-oriented, emotion-oriented, and avoidance-oriented coping. Alternative health-behavior measurement models compared a one-factor model for HB1–HB9 with a multidomain specification comprising physical activity based on HB1–HB4, dietary habits based on HB5–HB6, and a provisional sleep/screen factor based on HB7–HB8, with HB9 retained as a separate observed outcome representing overall routine regularity.

In the revised structural model, policy cognition and perceived stress were modeled as latent variables indicated by their respective items. Task-oriented, emotion-oriented, and avoidance-oriented coping were modeled as three correlated but distinct latent variables. Physical activity, dietary habits, weekday sleep duration, screen-time health score, and overall routine regularity were modeled as five separate outcomes.

Perceived stress was regressed on policy cognition. Each coping dimension was regressed on policy cognition and perceived stress. Each of the five health-behavior outcomes was regressed on policy cognition, perceived stress, task-oriented coping, emotion-oriented coping, and avoidance-oriented coping.

Three structural specifications were estimated. Model 1 was unadjusted and included all 1,513 participants. Model 2 adjusted for sex and rural–urban background and also included all 1,513 participants. Model 3 additionally adjusted for PE talent status, weekly school PE lessons, extracurricular physical activity, family exercise frequency, and parental encouragement and included 1,198 participants with complete valid covariate data. Categorical covariates were represented using dummy variables. The definitions, analytic representation, reference categories, and valid sample sizes of the covariates are reported in Supplementary Table S4.

Model fit was evaluated using χ^2^, degrees of freedom, χ^2^/df, the comparative fit index, the Tucker–Lewis index, the root mean square error of approximation, and the standardized root mean square residual (Hu and Bentler, 1999; Kline, 2016; Schreiber et al., 2006). Core standardized structural coefficients across the three model specifications are reported in Supplementary Table S5.

The final analysis-ready dataset did not contain reliable identifiers that allowed the school- and class-level nesting structure to be reconstructed. Consequently, the exact numbers of analytic school and class clusters, intraclass correlations, cluster-robust standard errors, and multilevel structural equation models could not be estimated retrospectively. The analyses therefore treated student records as individual observations. All findings were interpreted as concurrent cross-sectional statistical associations and not as evidence of temporal ordering, causal mediation, or intervention effectiveness.

The survey was conducted in one participating school. Verified class identifiers were not retained in the de-identified analysis-ready dataset; consequently, the exact number of participating classes, intraclass correlations, cluster-robust standard errors, and multilevel structural equation models could not be estimated retrospectively. The analyses therefore treated student records as individual observations. Unmodeled within-class dependence may have resulted in underestimated standard errors, and small coefficients close to conventional significance thresholds were interpreted cautiously.

### 2.5 Ethical considerations

This study involved human participants who were minors. It consisted of an anonymous, non-interventional, school-based questionnaire survey and involved no experimental manipulation.

Before data collection, the participating school conducted an administrative review and issued written authorization for the research team to conduct the survey and use the resulting de-identified data for academic research and publication. The authorization document was signed and stamped by the participating school on 9 July 2025. The school-level documentation described the project as anonymous, non-interventional, and minimal risk.

However, no approval or formal exemption was obtained from an independent institutional research ethics committee before data collection. No independent institutional body made a prospective determination that formal ethics review was not required. The participating school's authorization therefore constituted administrative permission and should not be interpreted as institutional ethics approval or exemption.

Written informed consent was obtained from a parent or legal guardian before student participation. The study purpose and questionnaire procedures were explained to students using age-appropriate language, and student assent was obtained before questionnaire completion. Participation was voluntary. Students were informed that they could decline or discontinue participation without penalty and that participation and responses would not affect their grades or school evaluation.

No directly identifying information, including names, student identification numbers, telephone numbers, or contact details, was collected. The data were de-identified and used exclusively for research purposes, and access to the electronic dataset was restricted to authorized members of the research team.

## 3 Results

### 3.1 Sample characteristics and measurement-model comparisons

The sample included 748 boys (49.4%) and 765 girls (50.6%). A total of 724 students (47.9%) had a rural background, and 789 students (52.1%) had an urban background. Sample characteristics are summarized in Table 2.

**Table 2:** Sample characteristics.

Variable	Category	*n*	%
Sex	Boys	748	49.4
	Girls	765	50.6
Self-reported rural–urban background	Rural background	724	47.9
	Urban background	789	52.1

Rural–urban background was an individual-level self-reported demographic characteristic and did not indicate the geographic location or institutional classification of the participating school.

Additional evaluation of the policy-cognition instrument supported an empirical one-factor representation in the present sample. The Kaiser–Meyer–Olkin value was 0.879, and Bartlett's test of sphericity was significant, χ^2^(10) = 3,921.564, *p* < 0.001. The first and second observed eigenvalues were 3.419 and 0.543, respectively, with the first factor accounting for 68.38% of the total variance. Parallel analysis supported retention of one factor, and principal-axis factor loadings ranged from 0.662 to 0.873.

In the confirmatory analysis, standardized policy-cognition loadings ranged from 0.668 to 0.867. Corrected item–total correlations ranged from 0.622 to 0.801, Cronbach's α was 0.882, composite reliability was 0.886, and average variance extracted was 0.609. These findings provide preliminary internal structural support for an empirical one-factor summary in this sample but do not establish that the five content facets are conceptually identical or that full construct validity has been demonstrated. Item-specific information is provided in Supplementary Table S1.

The original aggregate coping factor was not supported as a uniformly positive construct. Task-oriented coping loaded positively on the original aggregate factor (λ = 0.631), whereas emotion-oriented coping (λ = −0.813) and avoidance-oriented coping (λ = −0.708) loaded negatively.

The one-factor coping model showed poor fit, χ^2^(90) = 3,745.954, CFI = 0.705, TLI = 0.656, RMSEA = 0.164, and SRMR = 0.111. In contrast, the correlated three-factor coping model showed good fit, χ^2^(87) = 337.016, CFI = 0.980, TLI = 0.976, RMSEA = 0.044, and SRMR = 0.027.

The one-factor health-behavior model also showed poor fit, χ^2^(27) = 1,554.828, CFI = 0.758, TLI = 0.678, RMSEA = 0.193, and SRMR = 0.096. The multidomain specification showed substantially improved fit. χ^2^(17) = 201.894, CFI = 0.964, TLI = 0.941, RMSEA = 0.085, and SRMR = 0.049. However, the provisional two-item sleep/screen factor showed insufficient reliability. The revised structural analyses therefore treated physical activity, dietary habits, weekday sleep duration, screen-time health score, and overall routine regularity as five separate outcomes.

Fit indices for the alternative coping and health-behavior measurement models are summarized in Table 3.

**Table 3:** Comparison of alternative measurement models.

Construct and model	*N*	χ^2^	df	CFI	TLI	RMSEA	SRMR
Coping: one-factor model	1,513	3,745.954	90	0.705	0.656	0.164	0.111
Coping: correlated three-factor model	1,513	337.016	87	0.980	0.976	0.044	0.027
Health behavior: one-factor HB1–HB9 model	1,513	1,554.828	27	0.758	0.678	0.193	0.096
Health behavior: multidomain HB1–HB8 model	1,513	201.894	17	0.964	0.941	0.085	0.049

The multidomain health-behavior specification modeled physical activity using HB1–HB4, dietary habits using HB5–HB6, and a provisional sleep/screen factor using HB7–HB8; HB9 was retained as a separate observed measure of overall routine regularity.

Reliability and convergent-validity indices for the three coping dimensions are presented in Table 1. The exact wording, response options, and dimensional assignment of the coping items are provided in Supplementary Table S2. The complete health-behavior item mapping and analysis-ready scoring directions are provided in Supplementary Table S3.

### 3.2 Domain-specific structural models and covariate sensitivity analyses

All three structural models showed acceptable fit (Table 4). The unadjusted and sex/rural–urban-adjusted models included all 1,513 participants, whereas the extended-adjusted model included 1,198 participants with complete valid covariate data. Definitions, analytic representation, reference categories, and valid sample sizes for the covariates are presented in Supplementary Table S4. Core standardized structural coefficients across the three model specifications are reported in Supplementary Table S5.

**Table 4:** Fit indices for the structural sensitivity models.

Model	*N*	χ^2^	df	χ^2^/df	CFI	TLI	RMSEA	SRMR
Unadjusted	1,513	1,247.519	520	2.399	0.974	0.970	0.030	0.033
Sex and rural–urban adjusted	1,513	1,341.264	575	2.333	0.973	0.968	0.030	0.033
Extended covariate adjusted	1,198	1,826.641	989	1.847	0.968	0.961	0.027	0.043

Across all three specifications, policy cognition was negatively associated with perceived stress and positively associated with task-oriented coping. In the extended-adjusted model, policy cognition was negatively associated with perceived stress (β = −0.431, *p* < 0.001), positively associated with task-oriented coping (β = 0.286, *p* < 0.001), negatively associated with emotion-oriented coping (β = −0.076, *p* =0.022), and negatively associated with avoidance-oriented coping (β = −0.175, *p* < 0.001).

Perceived stress was negatively associated with task-oriented coping (β = −0.183, *p* < 0.001) and positively associated with emotion-oriented coping (β = 0.402, *p* < 0.001) and avoidance-oriented coping (β = 0.229, *p* < 0.001) in the extended-adjusted model.

In the fully adjusted model, perceived stress was negatively associated with physical activity (β = −0.246, *p* < 0.001), dietary habits (β = −0.167, *p* < 0.001), weekday sleep duration (β = −0.167, *p* < 0.001), screen-time health score (β = −0.183, *p* < 0.001), and overall routine regularity (β = −0.164, *p* < 0.001).

Task-oriented coping was positively associated with physical activity (β = 0.254, *p* < 0.001), dietary habits (β = 0.174, *p* < 0.001), weekday sleep duration (β = 0.157, *p* < 0.001), screen-time health score (β = 0.222, *p* < 0.001), and overall routine regularity (β = 0.192, *p* < 0.001).

Emotion-oriented coping was not independently associated with any of the five health-behavior outcomes. Avoidance-oriented coping was negatively associated with dietary habits (β = −0.213, *p* < 0.001) but was not significantly associated with physical activity, weekday sleep duration, screen-time health score, or overall routine regularity.

After extended adjustment, policy cognition was not significantly associated with physical activity, dietary habits, screen-time health score, or overall routine regularity. A small positive association remained between policy cognition and weekday sleep duration (β = 0.070, *p* = 0.041). Because cluster-adjusted standard errors were unavailable, this isolated small association close to the conventional significance threshold should be interpreted cautiously.

The fully adjusted standardized associations with the five health-behavior outcomes are presented in Table 5.

**Table 5:** Fully adjusted standardized associations with health-behavior outcomes.

Outcome	Policy cognition	Perceived stress	Task-oriented coping	Emotion-oriented coping	Avoidance-oriented coping
Physical activity	0.008, *p* = 0.798	−0.246^***^	0.254^***^	0.058, *p* = 0.210	−0.073, *p* = 0.089
Dietary habits	0.052, *p* = 0.120	−0.167^***^	0.174^***^	0.045, *p* = 0.361	−0.213^***^
Weekday sleep duration	0.070, *p* = 0.041	−0.167^***^	0.157^***^	0.015, *p* = 0.774	−0.087, *p* = 0.066
Screen-time health score	−0.019, *p* = 0.576	−0.183^***^	0.222^***^	0.062, *p* = 0.226	0.017, *p* = 0.715
Overall routine regularity	0.035, *p* = 0.307	−0.164^***^	0.192^***^	0.086, *p* = 0.096	0.048, *p* = 0.319

Standardized coefficients are reported. ^***^p < 0.001. The extended-adjusted model included sex, rural–urban background, PE talent status, school PE lessons, extracurricular physical activity, family exercise frequency, and parental encouragement.

The consistency and attenuation of the focal coefficients across the unadjusted, sex/rural–urban-adjusted, and extended-adjusted specifications can be examined in Supplementary Table S5.

## 4 Discussion

### 4.1 Summary of main findings

The revised analyses provided a more differentiated account of coping and health behavior. Task-oriented, emotion-oriented, and avoidance-oriented coping did not form a coherent uniformly positive factor, and the documented health-behavior outcomes could not be adequately represented by a single latent factor.

Perceived stress and task-oriented coping showed the most consistent associations across physical activity, dietary habits, weekday sleep duration, screen-time behavior, and overall routine regularity. Higher perceived stress was associated with less favorable scores across all five outcomes, whereas task-oriented coping was associated with more favorable scores. Emotion-oriented coping showed no independent associations after the other variables were considered. Avoidance-oriented coping was associated primarily with less favorable dietary habits.

A further important finding concerned policy cognition. Its direct associations with most health outcomes were substantially attenuated after adjustment for demographic, school-PE, prior-activity, and family-related variables. Policy cognition was more consistently associated with perceived stress and coping dimensions than with health behavior independently of these covariates.

Because all variables were measured concurrently, none of these associations establishes temporal direction or causal mediation.

### 4.2 Policy cognition as a policy-related subjective appraisal

The findings indicate that policy cognition was cross-sectionally associated with how students reported stress and coping in the PE examination context. Policy cognition should not be interpreted as an established causal pattern of associations through which policy reform changes health behavior. Its direct associations with most health outcomes were not robust to extended covariate adjustment.

The separate coping results are theoretically informative. Higher policy-cognition scores were associated with more task-oriented coping and with lower emotion-oriented and avoidance-oriented coping, whereas higher perceived stress showed the opposite pattern. These results support distinguishing the three coping dimensions rather than interpreting them as a single quantity of coping.

The stable associations of perceived stress and task-oriented coping with the five health outcomes are consistent with stress–coping perspectives, but the cross-sectional design does not establish whether stress preceded coping or health behavior (Lazarus and Folkman, 1984; Endler and Parker, 1990; Skinner and Zimmer-Gembeck, 2007; Sun et al., 2013; Zhu et al., 2021; Steare et al., 2023). Reciprocal relations remain possible.

### 4.3 . Practical and policy implications

The observed associations suggest that stress-related support and task-oriented coping skills warrant evaluation in future longitudinal and intervention studies. However, the present cross-sectional findings do not demonstrate that modifying policy cognition, perceived stress, or coping strategies would improve health behavior.

The attenuation of policy-cognition coefficients after covariate adjustment also indicates that policy communication should not be treated as a sufficient intervention by itself. School PE opportunities, previous physical-activity participation, and family support may be relevant contextual factors that should be incorporated into future program design and evaluation.

The findings should therefore be used to formulate testable intervention hypotheses rather than to recommend specific programs as already effective.

### 4.4 Limitations and future directions

Several limitations require emphasis. First, the cross-sectional design does not establish temporal ordering, causal mediation, reciprocal direction, or intervention effectiveness. Although the structural ordering was theory-informed, reverse and reciprocal associations remain possible.

Second, all principal variables were self-reported at one time point. Common-method variance, recall error, response-style associations, and social-desirability bias therefore cannot be ruled out. The earlier single-factor comparison based on the superseded four-factor model was not retained as evidence that all possible common-method variance was absent.

Third, the policy-cognition measure was a brief, context-specific, satisfaction-based instrument developed for the present study. The available analyses provided preliminary evidence concerning its internal structure and reliability, but the measure has not undergone comprehensive independent validation. The satisfaction-based response anchors do not directly assess objective policy knowledge, factual accuracy, or policy fairness, and the five content facets should not be interpreted as conceptually identical.

Fourth, the original aggregate coping model combined task-oriented, emotion-oriented, and avoidance-oriented coping even though the subscale indicators loaded in opposite directions. The revised analyses treated these dimensions separately, but the cross-sectional data cannot determine whether coping preceded or followed perceived stress or health behavior.

Fifth, the health-behavior questionnaire comprised several distinct outcomes rather than one homogeneous construct. Hb10 was excluded because no corresponding administered item, wording, response options, scoring rule, or documented source could be verified. The documented HB1–HB9 item mapping and analysis-ready scoring directions are reported in Supplementary Table S3. The original wording and response formats of HB5 and HB8 were not fully aligned; these features are reported transparently and were not modified retrospectively. Weekday sleep duration, screen-time health score, and overall routine regularity were therefore analyzed separately.

Sixth, the extended-adjusted model was restricted to 1,198 participants with complete valid contextual data. Grade could not be included because no valid grade variable was retained in the final analysis-ready dataset, and it was not reconstructed from birth year or other fields. Complete-case analysis may also have affected the composition of the extended-adjusted sample.

Seventh, the final analysis-ready dataset did not contain reliable identifiers that allowed the school- and class-level nesting structure to be reconstructed. Consequently, the exact numbers of analytic school and class clusters, intraclass correlations, cluster-robust standard errors, and multilevel models could not be estimated. Unmodeled within-school or within-class dependence may have resulted in underestimated standard errors and overly liberal significance tests. This limitation is particularly relevant to small coefficients close to the conventional significance threshold, including the adjusted association between policy cognition and weekday sleep duration.

Finally, the participants were drawn from a specific regional school context. The findings should not be generalized automatically to other regions, examination systems, school types, or age groups. Replication using multi-school, multi-region, longitudinal, objective-behavior, and cluster-aware designs is required.

## 5 Conclusion

This cross-sectional study found that perceived stress and task-oriented coping were consistently associated with physical activity, dietary habits, weekday sleep duration, screen-time behavior, and overall routine regularity. Emotion-oriented coping was not independently associated with the documented outcomes, whereas avoidance-oriented coping was negatively associated primarily with dietary habits.

Direct associations between policy cognition and most health outcomes were substantially attenuated after adjustment for sex, rural–urban background, PE talent status, school PE lessons, extracurricular physical activity, family exercise frequency, and parental encouragement. Policy cognition was more consistently associated with perceived stress and coping dimensions than with health behaviors independently of these covariates.

These findings represent concurrent statistical associations and do not establish temporal ordering, causal mediation, or policy-associationiveness. Longitudinal, cluster-aware, and intervention studies are required before causal or policy-effectiveness conclusions can be drawn.

## Data Availability

The datasets presented in this study can be found in online repositories. The names of the repository/repositories and accession number(s) can be found in the article/Supplementary material.
