# Supplementary material for: Policy cognition, perceived stress, coping dimensions, and adolescent health behaviors under physical education examination reform: a cross-sectional SEM study
Source: Front Psychol. 2026 Sep 22;17:1896758. doi: 10.3389/fpsyg.2026.1896758 (PMC13638618; doi:10.3389/fpsyg.2026.1896758)
Supplement: Supplementary file 1 [file Supplementary_File_1.docx]

**Supplementary Materials**

**Manuscript title:** Policy Cognition, Perceived Stress, Coping Dimensions, and Adolescent Health Behaviors under Physical Education Examination Reform: A Cross-Sectional SEM Study

**Supplementary Table S1.** Exact wording, content facets, response options, and standardized loadings of the policy-cognition items
**Supplementary Table S2.** Exact wording, response options, and dimensional assignment of the coping items
**Supplementary Table S3.** Exact wording, original response options, analysis-ready scoring direction, and outcome assignment of the nine documented health-behavior items
**Supplementary Table S4.** Covariate definitions, analytic representation, reference categories, and valid sample sizes
**Supplementary Table S5.** Core standardized structural coefficients across the unadjusted and covariate-adjusted models

Supplementary Table S1. Exact wording, conceptual content, and response options of the policy-cognition items

| Code | Content facet | Exact Chinese item | English translation | Response options | Standardized CFA loading |
| --- | --- | --- | --- | --- | --- |
| PCog1 | Rule clarity | 我清楚云南省体育中考方案的评分标准与规则 | I clearly understand the scoring standards and rules of the Yunnan PE entrance-examination scheme. | 1 = very dissatisfied to 7 = very satisfied | 0.786 |
| PCog2 | Reasonableness and necessity | 我认为体育中考纳入总分是合理且必要的 | I consider the inclusion of PE in the total entrance-examination score to be reasonable and necessary. | 1 = very dissatisfied to 7 = very satisfied | 0.776 |
| PCog3 | Preparedness and school guidance | 我知道如何准备体育中考，学校和老师也提供明确指导 | I know how to prepare for the PE entrance examination, and the school and teachers provide clear guidance. | 1 = very dissatisfied to 7 = very satisfied | 0.867 |
| PCog4 | Health and developmental value | 我认为体育中考对我的身体健康与成长非常重要 | I consider the PE entrance examination important for my physical health and development. | 1 = very dissatisfied to 7 = very satisfied | 0.792 |
| PCog5 | Social salience | 我周围的人（家长、同学）常讨论体育中考政策 | People around me, including parents and classmates, often discuss the PE entrance-examination policy. | 1 = very dissatisfied to 7 = very satisfied | 0.668 |

**Note.** All items were rated from 1 (very dissatisfied) to 7 (very satisfied). Higher mean scores represented a more favorable satisfaction-based subjective appraisal of the PE entrance-examination policy rather than objectively more accurate policy knowledge. The five content facets were modeled as indicators of one empirical policy-cognition factor in the present sample; this specification does not imply that the facets are conceptually identical or that full construct validity has been established. Cronbach’s α was 0.882, composite reliability was 0.886, and average variance extracted was 0.609.

**Supplementary Table S2. Exact wording, response options, and dimensional assignment of the coping items**

| Code | Dimension | Exact Chinese item | English translation | Response options |
| --- | --- | --- | --- | --- |
| C1 | Task-oriented | 当面对体育考试压力时，我制定计划并按部就班地完成任务。 | When facing PE examination pressure, I make a plan and complete tasks step by step. | 1 = never to 5 = almost always |
| C2 | Task-oriented | 我努力找到解决考试问题的具体方法。 | I try to find concrete ways to solve examination-related problems. | 1 = never to 5 = almost always |
| C3 | Task-oriented | 我尝试合理安排时间来应对体育测试。 | I try to manage my time appropriately to cope with the PE test. | 1 = never to 5 = almost always |
| C4 | Task-oriented | 我关注完成目标而非担忧问题。 | I focus on completing the goal rather than worrying about the problem. | 1 = never to 5 = almost always |
| C5 | Task-oriented | 我主动寻求资源来帮助我解决难题。 | I actively seek resources to help me solve difficulties. | 1 = never to 5 = almost always |
| C6 | Emotion-oriented | 我感到紧张并容易情绪失控。 | I feel tense and easily lose emotional control. | 1 = never to 5 = almost always |
| C7 | Emotion-oriented | 我责备自己为什么没准备好。 | I blame myself for not being adequately prepared. | 1 = never to 5 = almost always |
| C8 | Emotion-oriented | 我变得烦躁易怒，甚至和家人争吵。 | I become irritable and may even argue with family members. | 1 = never to 5 = almost always |
| C9 | Emotion-oriented | 我对自己失望，觉得自己不行。 | I feel disappointed in myself and believe that I am incapable. | 1 = never to 5 = almost always |
| C10 | Emotion-oriented | 我常常因情绪低落而放弃训练或锻炼。 | I often stop training or exercising because of low mood. | 1 = never to 5 = almost always |
| C11 | Avoidance-oriented | 我尽量不去想体育考试的事情。 | I try not to think about the PE examination. | 1 = never to 5 = almost always |
| C12 | Avoidance-oriented | 我用打游戏或刷手机来逃避压力。 | I use gaming or smartphone browsing to escape from stress. | 1 = never to 5 = almost always |
| C13 | Avoidance-oriented | 我会故意少上体育课或找借口逃避训练。 | I deliberately attend fewer PE classes or make excuses to avoid training. | 1 = never to 5 = almost always |
| C14 | Avoidance-oriented | 我选择与朋友闲聊以避免思考考试问题。 | I chat with friends to avoid thinking about examination-related problems. | 1 = never to 5 = almost always |
| C15 | Avoidance-oriented | 我会躲避与考试有关的话题或人。 | I avoid examination-related topics or people. | 1 = never to 5 = almost always |

**Note.** Responses ranged from 1 (never) to 5 (almost always). Standardized loading ranges were 0.648–0.858 for task-oriented coping, 0.758–0.855 for emotion-oriented coping, and 0.724–0.771 for avoidance-oriented coping. Cronbach’s α values were 0.870, 0.898, and 0.861; composite reliability values were 0.872, 0.898, and 0.862; and average variance extracted values were 0.579, 0.638, and 0.555, respectively.

**Supplementary Table S3. Exact wording, original response options, analysis-ready scoring direction, and outcome assignment of the nine documented health-behavior items**

| Code | Exact Chinese item | English translation | Original response options | Final outcome and analysis-ready scoring |
| --- | --- | --- | --- | --- |
| HB1 | 过去7天内，你每周进行了多少次中等强度身体活动（如快走、骑车）？ | During the past 7 days, how many times per week did you engage in moderate-intensity physical activity, such as brisk walking or cycling? | 1=从不；2=每周1次；3=每周2–3次；4=每周4–5次；5=每天 | Physical activity; higher scores indicate greater activity frequency |
| HB2 | 过去7天内，你每次中等强度活动平均持续多长时间？ | During the past 7 days, how long did each moderate-intensity activity session usually last? | 1=<15分钟；2=15–30分钟；3=31–45分钟；4=46–60分钟；5=>60分钟 | Physical activity; higher scores indicate longer activity duration |
| HB3 | 过去7天内，你每周进行了多少次剧烈运动（如跑步、篮球）？ | During the past 7 days, how many times per week did you engage in vigorous physical activity, such as running or basketball? | 1=从不；2=每周1次；3=每周2–3次；4=每周4–5次；5=每天 | Physical activity; higher scores indicate greater activity frequency |
| HB4 | 过去7天内，你每次剧烈运动平均持续多长时间？ | During the past 7 days, how long did each vigorous activity session usually last? | 1=<15分钟；2=15–30分钟；3=31–45分钟；4=46–60分钟；5=>60分钟 | Physical activity; higher scores indicate longer activity duration |
| HB5 | 你通常每天吃几顿有蔬菜的正餐？ | How often do you usually eat main meals that include vegetables? | 1=从不；2=每周1–2次；3=每周3–4次；4=几乎每天；5=每天三餐都有 | Dietary habits; higher scores indicate more frequent vegetable intake |
| HB6 | 你通常每天饮用含糖饮料的频率是？ | How often do you usually consume sugar-sweetened beverages? | 1=从不；2=偶尔；3=每周1–2次；4=每周3–4次；5=每天 | Dietary habits; the revised analysis used the archived health-oriented analysis field, in which higher values indicated less frequent sugar-sweetened beverage consumption. No additional reverse scoring was applied during the revised reanalysis |
| HB7 | 你通常工作日睡眠时间为多少小时？ | How many hours do you usually sleep on weekdays? | 1=<5小时；2=5–6小时；3=6–7小时；4=7–8小时；5=>8小时 | Weekday sleep duration; higher scores indicate longer reported weekday sleep duration |
| HB8 | 你是否每天使用电子产品时间超过2小时？ | How much time do you usually spend using electronic devices each day? | 1=<1小时；2=1–2小时；3=2–3小时；4=3–4小时；5=>4小时 | Screen-time health score; the revised analysis used the archived reverse-oriented analysis field, in which higher values indicated less daily electronic-device time. No additional reverse scoring was applied during the revised reanalysis |
| HB9 | 你是否每天都有规律的饮食与作息习惯？ | How regular are your daily dietary and routine habits? | 1=非常不规律；2=较不规律；3=一般；4=较规律；5=非常规律 | Overall routine regularity; higher scores indicate greater regularity |

**Note.**Physical activity was calculated as the mean of HB1–HB4. Dietary habits were calculated from HB5 and the archived health-oriented analysis field for HB6. HB7, HB8, and HB9 were analyzed separately as weekday sleep duration, screen-time health score, and overall routine regularity. No Hb10 item is reported because no corresponding administered questionnaire item, wording, response options, scoring rule, or documented source could be verified. The original Chinese wording and response categories are reproduced as documented in the administered questionnaire. HB5 and HB8 contained minor wording–response-format inconsistencies in the original instrument; no post hoc wording correction was made.

**Supplementary Table S4. Covariate definitions, analytic representation, reference categories, and valid sample sizes**

| Covariate | Original categories | Analytic representation | Reference category | Valid N |
| --- | --- | --- | --- | --- |
| Sex | Boy; Girl | Female indicator | Boy | 1,513 |
| Rural–urban background | Rural; Urban | Urban-background indicator | Rural background | 1,513 |
| PE talent status | Yes; No | PE-talent indicator | No | 1,211 |
| Weekly school PE lessons | 1 lesson; 2 lessons; 3 or more lessons | Dummy variables | 3 or more lessons | 1,214 |
| Weekly extracurricular physical activity | <1 hour; 1–3 hours; >3 hours | Dummy variables | >3 hours | 1,208 |
| Family exercise frequency | Rarely; Occasionally; Often; Every day | Dummy variables | Every day | 1,206 |
| Parental encouragement | Very discouraging; Discouraging; Neutral; Encouraging; Very encouraging | Dummy variables | Very encouraging | 1,212 |

**Note.** Sex and rural–urban background were available for all 1,513 participants. A total of 1,198 participants had complete valid data for all extended covariates and were included in the extended-adjusted model. The extended-adjusted model used complete-case analysis across all included extended covariates. Grade was not included because no valid grade variable was retained in the final analysis-ready dataset, and it was not reconstructed from birth year or other fields.

**Supplementary Table S5. Core standardized structural coefficients across the unadjusted and covariate-adjusted models**

| Outcome | Predictor | Unadjusted β | Sex/rural–urban adjusted β | Extended-adjusted β | Extended p |
| --- | --- | --- | --- | --- | --- |
| Perceived stress | Policy cognition | −0.421 | −0.409 | −0.431 | <.001 |
| Task-oriented coping | Policy cognition | 0.387 | 0.386 | 0.286 | <.001 |
| Task-oriented coping | Perceived stress | −0.127 | −0.127 | −0.183 | <.001 |
| Emotion-oriented coping | Policy cognition | −0.077 | −0.076 | −0.076 | .022 |
| Emotion-oriented coping | Perceived stress | 0.418 | 0.421 | 0.402 | <.001 |
| Avoidance-oriented coping | Policy cognition | −0.156 | −0.159 | −0.175 | <.001 |
| Avoidance-oriented coping | Perceived stress | 0.227 | 0.247 | 0.229 | <.001 |
| Physical activity | Policy cognition | 0.084 | 0.076 | 0.008 | .798 |
| Physical activity | Perceived stress | −0.253 | −0.224 | −0.246 | <.001 |
| Physical activity | Task-oriented coping | 0.344 | 0.334 | 0.254 | <.001 |
| Physical activity | Emotion-oriented coping | 0.056 | 0.078 | 0.058 | .210 |
| Physical activity | Avoidance-oriented coping | −0.008 | −0.053 | −0.073 | .089 |
| Dietary habits | Policy cognition | 0.103 | 0.101 | 0.052 | .120 |
| Dietary habits | Perceived stress | −0.150 | −0.142 | −0.167 | <.001 |
| Dietary habits | Task-oriented coping | 0.217 | 0.214 | 0.174 | <.001 |
| Dietary habits | Emotion-oriented coping | 0.045 | 0.051 | 0.045 | .361 |
| Dietary habits | Avoidance-oriented coping | −0.174 | −0.186 | −0.213 | <.001 |
| Weekday sleep duration | Policy cognition | 0.102 | 0.100 | 0.070 | .041 |
| Weekday sleep duration | Perceived stress | −0.156 | −0.146 | −0.167 | <.001 |
| Weekday sleep duration | Task-oriented coping | 0.206 | 0.203 | 0.157 | <.001 |
| Weekday sleep duration | Emotion-oriented coping | −0.002 | 0.003 | 0.015 | .774 |
| Weekday sleep duration | Avoidance-oriented coping | −0.064 | −0.078 | −0.087 | .066 |
| Screen-time health score | Policy cognition | 0.009 | 0.004 | −0.019 | .576 |
| Screen-time health score | Perceived stress | −0.201 | −0.182 | −0.183 | <.001 |
| Screen-time health score | Task-oriented coping | 0.222 | 0.215 | 0.222 | <.001 |
| Screen-time health score | Emotion-oriented coping | 0.026 | 0.042 | 0.062 | .226 |
| Screen-time health score | Avoidance-oriented coping | 0.063 | 0.034 | 0.017 | .715 |
| Overall routine regularity | Policy cognition | 0.047 | 0.041 | 0.035 | .307 |
| Overall routine regularity | Perceived stress | −0.188 | −0.172 | −0.164 | <.001 |
| Overall routine regularity | Task-oriented coping | 0.182 | 0.174 | 0.192 | <.001 |
| Overall routine regularity | Emotion-oriented coping | 0.059 | 0.075 | 0.086 | .096 |
| Overall routine regularity | Avoidance-oriented coping | 0.072 | 0.046 | 0.048 | .319 |

**Note.** Standardized coefficients for the focal structural paths are reported. The basic-adjusted model included sex and rural–urban background. The extended-adjusted model additionally included PE talent status, school PE lessons, extracurricular physical activity, family exercise frequency, and parental encouragement. The extended model included 1,198 complete-valid cases. Covariate-specific coefficients are not displayed in this summary table. Because reliable school and class identifiers were unavailable, standard errors and p values were not cluster-adjusted.
